# Simultaneous Indoor Pedestrian Localization and House Mapping Based on Inertial Measurement Unit and Bluetooth Low-Energy Beacon Data

**DOI:** 10.3390/s20174742

**Published:** 2020-08-22

**Authors:** Jesus D. Ceron, Felix Kluge, Arne Küderle, Bjoern M. Eskofier, Diego M. López

**Affiliations:** 1 Telematics Engineering Research Group, Telematics Department, Universidad Del Cauca (Unicauca), Popayán 190002, Colombia; jesusceron@unicauca.edu.co; 2 Machine Learning and Data Analytics Lab, Computer Science Department, Friedrich-Alexander University, Erlangen-Nürnberg (FAU), 91052 Erlangen, Germany; felix.kluge@fau.de (F.K.); arne.kuederle@fau.de (A.K.)

**Keywords:** indoor localization, indoor tracking, SLAM, particle filter

## Abstract

Indoor location estimation is crucial to provide context-based assistance in home environments. In this study, a method for simultaneous indoor pedestrian localization and house mapping is proposed and evaluated. The method fuses a person’s movement data from an Inertial Measurement Unit (IMU) with proximity and activity-related data from Bluetooth Low-Energy (BLE) beacons deployed in the indoor environment. The person’s and beacons’ localization is performed simultaneously using a combination of particle and Kalman Filters. We evaluated the method using data from eight participants who performed different activities in an indoor environment. As a result, the average participant’s localization error was 1.05 ± 0.44 m, and the average beacons’ localization error was 0.82 ± 0.24 m. The proposed method is able to construct a map of the indoor environment by localizing the BLE beacons and simultaneously locating the person. The results obtained demonstrate that the proposed method could point to a promising roadmap towards the development of simultaneous localization and home mapping system based only on one IMU and a few BLE beacons. To the best of our knowledge, this is the first method that includes the beacons’ data movement as activity-related events in a method for pedestrian Simultaneous Localization and Mapping (SLAM).

## 1. Introduction

Based on the concept of ubiquitous computing introduced by Mark Weiser in 1990 [1], new research fields have emerged, such as the internet of things, ambient intelligence, smart homes, smart spaces, and ambient assisted living. The main purpose of those fields is to provide context-based assistance to the user. To provide such support, contextual information such as the location and the activity that a person is carrying out is useful. With continuous technological advances, new sensors, devices, and communication protocols have created new opportunities for obtaining data that allows for the estimation of such contextual information [2].

Global Navigation Satellite Systems (GNSS) such as Galileo, developed by the European Union and the Global Positioning System (GPS) developed by the United States, are widely known and used. This is because these systems can accurately track worldwide positions with small and cheap hardware. However, when the signals from the satellites come into contact with the surfaces of the indoor environments such as roofs or walls, the localization accuracy degrades. Besides, the interaction of the signals with fixed and mobile obstacles in indoor environments produces undesirable effects such as signal reflection and absorption. Therefore, these systems are not suitable for obtaining a location in indoor settings, where people spend most of their daily time [3,4]. To overcome these limitations, Indoor Positioning Systems (IPS) have been developed.

IPSs can be classified as range-based or range-free [5]. Range-based IPS involves the use of signal emitters and receiver devices. The device to be located is usually a portable device carried by the person (e.g., smartphone). Its location is obtained by estimating the distance (range) between it and other emitter/receiver devices deployed in the indoor environment. The methods commonly used for this purpose are trilateration, lateration, and arrival angle. These methods are based on techniques such as time of arrival (TOA), arrival time difference (TDOA), angle of arrival (AOA), and received signal strength (RSS).

Range-free IPSs do not require a continuous estimation of a distance between signal emitting and receiving devices. Three common range-free IPS are based on fingerprinting, vision analysis, and pedestrian dead reckoning (PDR) [5,6,7]. Fingerprinting has two phases. In its offline phase, the indoor environment is usually divided into cells. Measurements of the signal broadcasted for one or more emitting devices are collected in each cell by a receiver such as a smartphone and stored in a database. In the online phase, the receiving device’s position is estimated by comparing the received signal with the measurements previously stored in the offline phase. Vision analysis-based methods analyses images for detecting relevant characteristics of the indoor environment that allow the estimation of the person position. Finally, PDR makes use of movement information collected from IMUs placed on the person’s body to estimate their relative position to a starting point.

There are several IPSs, but each has its own set of advantages and disadvantages. The choice of an IPS should be based on the specific characteristics of the indoor environment, the desirable precision, budget constraints, obtrusiveness, system complexity, robustness, and privacy [2,6,7,8].

All range-based IPS require the configuration, deployment, and maintenance of the devices necessary for its correct operation, which means that its complexity is high. TOA and TDOA require expensive equipment for the synchronization of the emitters and receivers. AOA does not require time synchronization. However, it requires complex and expensive hardware compared to RSS [2,6]. RSS-based methods can be implemented with low-cost devices such as WiFi or Bluetooth antennas. Nonetheless, the RSS is affected by noise, attenuation, and fluctuation. To solve that problem, different techniques have been proposed in the literature, such as the filtering of the RSS signal using different variants of the Kalman filter and particle filters [9,10].

Regarding the range-free methods, the need to gather an offline map upfront increases the complexity. This is more critical, as a new offline map would be required whenever the environment is changed. That means that a fingerprinting-based IPS must include special methods to provide robustness. Computer vision-based methods are considered a sensitive solution because they affect the privacy of people in their houses. In contrast, PDR is considered a low-cost method since only one IMU on the foot or hip is required. Therefore, it is considered an unobtrusive and easy to use approach. Moreover, in principle, PDR does not need data from external devices to estimate the person’s position. However, it suffers from cumulative errors due to the integration of noisy motion data that make it unfeasible for estimating location over long periods [7].

Some IPS use hybrid approaches, a combination of range-based, and range-free IPS methods. Among them, the combination of PDR and range-based methods is recognized as a suitable approach specifically for indoor pedestrian localization, since the aim of that combination is to correct the accumulative error in PDR by closing the loop (to identify that the person has returned to a known location) [11]. Therefore, strategies that reduce the complexity of configuring, deploying, and maintaining the devices are required. Another key attribute of an IPS is the robustness. This is the capacity of the IPS to adapt automatically to changes that may occur in the indoor environment since an indoor environment can be considered a dynamic system [5,6].

Simultaneous Localization and Mapping (SLAM) usually addresses the PDR cumulative error issue by fusing the motion data from the IMU and observing landmarks [12]. The landmarks are well-defined markers or objects in the indoor environment, such as walls, corridors, stairs, or emitter/receiver devices. By using SLAM, no prior map or building layout is required, since it allows the construction of the map (mapping) while a person is moving freely and continuously tracked inside the indoor environment. The map is continually updated based on the trajectories of the person and the observation of landmarks. This means that with SLAM, the indoor environment is considered a dynamic system. A detailed explanation of SLAM applied in the context of human indoor localization is given in Appendix A.

Since 2012 when the Bluetooth Low Energy specification was released, the use of devices such as Bluetooth Low Energy (BLE) beacons has gained considerable popularity in the IoT-related fields thanks to its small size, low cost, and low energy consumption [13]. A useful characteristic of beacons is that they can integrate accelerometer, barometer, and light sensors, thus expanding opportunities for collecting more contextual information. For example, the activities that a person carries out at home are usually related to the use of particular objects (TV control, broom, toilet lid, etc.). The detection of movement of these objects, along with the detection of proximity to them, constitutes a source of useful information to determine the activity that the person is performing. At the same time, that information can be used as a landmark in SLAM.

This paper presents a novel method for Simultaneous Pedestrian Indoor Localization and house mapping based on human movement and activity-related data. The movement data is acquired from an IMU. The positioning estimates of PDR are improved by fusing them with proximity and activity-related data from BLE beacons. The proposed method was designed to reduce configuration, deployment, and maintenance efforts, and to be capable of adapting to changes in the indoor environment. These features make the proposed method promising to obtain accurate Indoor localization and activity recognition as part of an IPS in a smart home or an ambient assisted living scenario.

## 2. Related Work

The most common devices used as landmarks for SLAM have been range lasers scanners and cameras [5]. However, both of them are not suitable for indoor pedestrian localization due to privacy and obtrusiveness concerns. The works listed in Table 1 describe methods that implement pedestrian SLAM using PDR only or a combination of PDR and other range-based methods.

In Gu et al. [14], a building structure-based method for indoor pedestrian SLAM in which the landmarks were the corridors and corners of the indoor environment is proposed. Thus, no external devices are needed. The recognition of the landmarks was done based on the shape of the person’s trajectory. The position error obtained in their evaluation was 1.34 m in an office scenario of size 20 m × 20 m and 0.8 m in a library scenario of size 70 m × 70 m. These results make evident that the building structure’s complexity affects the position error obtained. ActionSLAM [15] used a similar approach, but in this case, the landmarks were specific actions performed by the person, such as sitting, standing, open/close door, or windows. Although the position error achieved for that method was 1.16 ± 0.07 m, the person must wear three IMUs: one in the foot for PDR, one on the hip for sitting and standing detection, and other in the wrist for hand actions detection. Also, the recognition of each activity must be carried out for an external set of algorithms. In the two previous works, the localization precision depends on both the intrinsic characteristics of the indoor environment and the frequency in which the landmarks are revisited.

FootSLAM, in conjunction with PlaceSLAM [16], implements a Rao-Blackwellized Particle Filter, where each particle contains an instance of the person trajectory and its related map. A foot-mounted IMU captured inertial data. The PDR open-loop position error was corrected when the proximity to someplace, such as corridors or doors, is detected by reading RFID tags placed on them. A position error of 2 m at two reference points was achieved. WiFi GraphSLAM [17] used RSS measurements to estimate the distance between the person and WiFi antennas, instead of using proximity data collected when the person interacts with RFID tags. The system was evaluated in a 60 m × 10 m area and the position error obtained was 2.23 ± 1.25 m.

A Graph-based optimization method that solves the SLAM problem is proposed in Zuo et al. [18]. It combines PDR and fingerprinting. That method was evaluated using 48 BLE beacons in an office context while one person holds one smartphone. The beacons were installed approximately evenly covering the complete area (90 m × 37 m). The positioning error was 3.25 m. The mean positioning error of the beacons was 1.27 m. A second evaluation was performed using only 24 beacons. In that case, the position error was 4.69 m, and the mean position error of the beacons was 2.26 m.

## 3. Dataset

**Figure 1:**
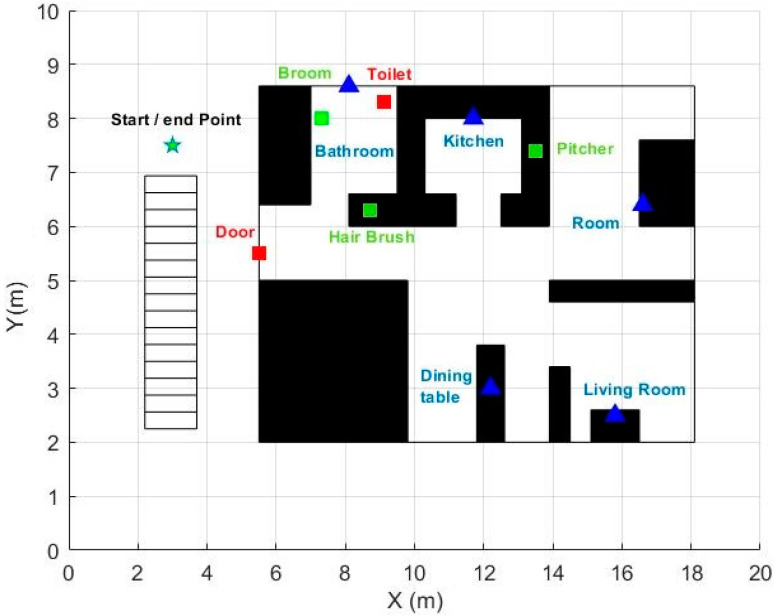
Map of the apartment. Blue triangles represent stationary beacons, red squares represent active beacons, and green squares represent mobile beacons.

An apartment was simulated in a room of the Machine Learning and Data Analytics lab at the University of Erlangen-Nuremberg, Germany (Figure 1). Eight participants (mean age: 29.4 ± 3.7 years) carried out the activities described in Table 2. Human movement, proximity, and action-related data were collected for each participant. Each session had an average duration of 6.21 ± 1.17 min.

### 3.1. Human Movement Data

Each participant used four Inertial Measurement Units - IMUs (Portabiles GmbH, Erlangen, Germany https://portabiles.de/) placed on each wrist and foot. The IMUs continuously communicate to synchronize their clocks. The sampling frequency used was 204.8 Hz, and the variables collected were acceleration (range: ± 16 g) and angular velocity (range: ± 2000 DPS).

A smartphone (Motorola G6 Plus) was carried in one of the participant’s pockets and also collected acceleration (range: ± 8 g) and angular velocity (range: ± 2000 DPS) data at a sampling frequency of 204.8 Hz. The data collected from the IMUs and the smartphone was synchronized using a python script that matched each data point’s timestamps. All sessions were recorded by video using a video camera. The videos were used as a reference for the evaluation process.

### 3.2. Proximity and Movement Object Data

BLE beacons are small devices that emit a BLE signal received and interpreted by devices such as a smartphone. Ten beacons were deployed in the room (red and green squares and blue triangles in Figure 1). The configuration and deployment were done following the recommendations of Castillo-Cara et al. [19]. They studied the beacon set up parameters such as transmission power, density, and topology. They recommended splitting the target area into sectors, keeping gaps between them to avoid a beacons’ signal overlapping. To this end, they suggest using low or medium transmission power.

The beacons used in the data collection (Estimote Proximity Beacons, Estimote Inc, San Francisco, CA, USA, https://estimote.com/) have built-in accelerometers. They were set up to send the raw acceleration data within telemetry packets (TLM packets) at a frequency of 10 Hz. Each TLM packet contains the universally unique identifier (UUID) of the beacon that emits it, three-axis acceleration raw data, the movement state of the beacon (moving or standing still), and the duration of the current state of movement. Along with each packet, the received signal strength indicator (RSSI) was received as an indicator of proximity. The transmission power was set at −20 dBm, with which the manufacturer indicates that a range of around 3.5 m radius is reached. A mobile application for Android devices was developed to receive the TLM packets from the ten beacons. The application was executed in the background during data collection on the smartphone carried in the participant’s pocket.

**Figure 2:**
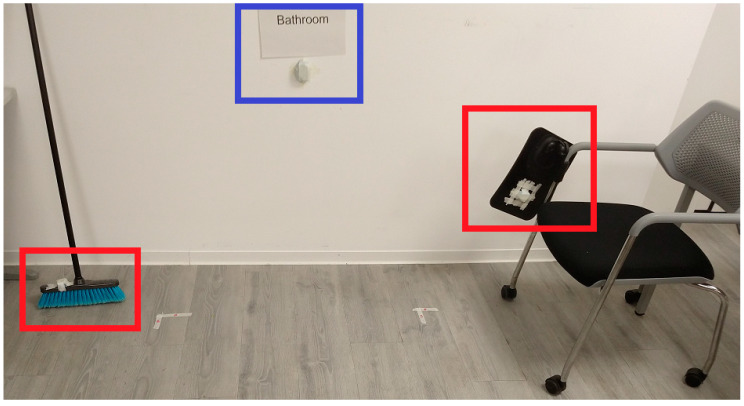
Example of types of beacons. Beacons in red squares are active. The beacon in the blue square is stationary.

Beacons were categorized as stationary, active, and mobile. Stationary beacons are meant to remain stationary since the participants do not interact with them. Consequently, the movement data of these five beacons are not used, only proximity data. The five stationary beacons are located in each apartment’s rooms (blue triangles in Figure 1), and all are at the height of approximately one meter from the floor. The remaining five beacons, red and green squares in Figure 1, are considered ‘active’ beacons because their movement indicates certain activity related to the object to which they are attached. For example, a movement of the beacon attached to the toilet lid (accompanied by proximity detection to it) could indicate the action of using the toilet (Figure 2). Finally, three of the five active beacons are considered ‘mobile’ (green boxes in Figure 1). These beacons are attached to objects that can change their position when used (Broom, pitcher, and hairbrush).

## 4. Proposed Method

**Figure 3:**
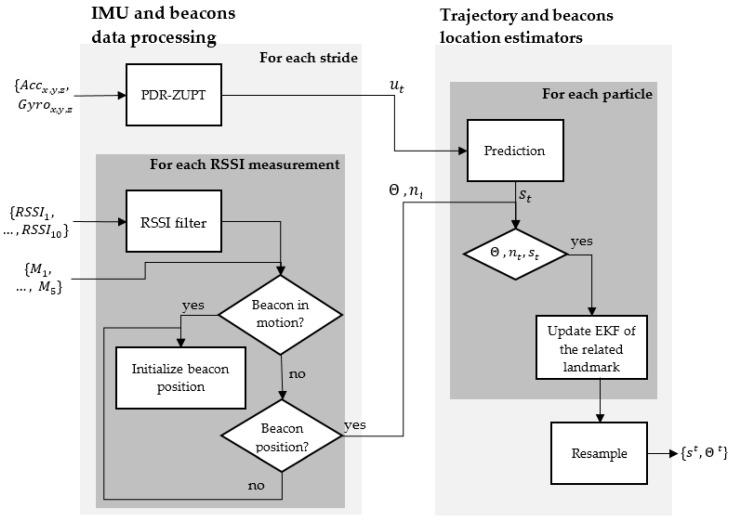
The proposed method for Simultaneous Pedestrian Indoor Localization and house mapping.

The proposed method for simultaneous pedestrian indoor localization and house mapping (Figure 3) is based on the Fast-SLAM algorithm [20]. The first block contains the logic proposed to process the data of the IMU and the beacons to obtain the necessary input variables required in the second block, which is responsible for estimating the person’s position and the position of the beacons. As in the original Fast-SLAM proposal, the estimation of the trajectory is done using a particle filter and, the estimation of the position of each landmark (beacon in this case) is made through the use of an Extended Kalman Filter (EKF). Each of the components of the proposed method is described below.

### 4.1. PDR-ZUPT

Foot-mounted PDR, together with a zero velocity update (ZUPT) algorithm, has been the most widely and successful method used to mitigate the intrinsic drift of PDR [21]. In this work, only the data taken from the IMU placed on the left foot were used. In this component, our method previously published for the strides’ detection and the estimation of their length and orientation was used [22]. The result is a measurement of movement $\;u_{t}$, which consists of an estimate of the person’s new position represented by a point on the x-y-z coordinate plane.

### 4.2. RSSI Filter

TLM packets sent from beacons are received by the smartphone. Along with the received packet, the smartphone receives the Received Signal Strength Indicator (RSSI). The relationship between the RSSI and the distance between the emitter beacon and the smartphone is given by the Log-distance path loss model:

(1) $$RSSI=10n\log_{10}\left(\frac{d}{d_{1m}}\right)+RSSI_{1m},$$

For calibration purposes, usually, a distance of 1 m between the emitter and receiver is taken as a reference point, that way,$\;d_{1m}=1\;m$, and $RSSI_{1m}$ equals the RSSI measured at 1 m. In ideal conditions, given any RSSI value, the exact distance between the beacon and the smartphone could be calculated with Equation (5). However, it is widely known that this relationship is not exact, mainly due to multipath propagation phenomena. Furthermore, the BLE signal is broadcasted on 2 MHz bandwidth channels in the 2.4 GHz band, causing the BLE signal to be affected by fading, even more so than WIFI signals [23]. Hence, before calculating the distance from the received RSSI readings, they are filtered with a one-dimensional Kalman Filter. The filter design followed the following steps:

(1) Choice of the state variable. The state variable is the signal to be filtered:
  (2) x=RSSI values
(2) Establish the parameters for the prediction phase. Two equations compose the one-variable Kalman filter prediction step:
  (3) $$\bar{x}=x+dx$$
  (4) $$\bar{P}=P+Q$$

The current state $\bar{x}$ equals the previous state (*x*) plus a value given by the process model (dx), a model describing how the state variable changes from the previous state to the current state. It is not possible to establish a mathematical function that estimates the current RSSI value based on the previous one. Therefore, it will be assumed that the current value will be equal to the previous one by  dx=0.

The variance of the current state $\bar{P}$ depends on the variance of the previous state (P) plus the uncertainty (variance) that the process model introduces, known as the process noise  (Q). In the beginning, P is unknown. It is then assumed that P is a large value indicating a lot of uncertainty in the initial estimate. Therefore, its initial value was set to $\;P={\left(24\;\mathrm{dBm}\right)}^{2}$. More precisely, P was initialized as the double of the variance of the z measurements. The process noise was established as $Q={\left(0.55\;\mathrm{dBm}\right)}^{2}$ empirically:

(3) Implement the update phase. There are four equations in this step:
  (5) $$y=z+\bar{x}$$
  (6) $$K=\frac{\bar{P}}{\bar{P}+R}$$
  (7) $$x=\bar{x}+Ky$$
  (8) $$P=\left(1-K\right)\bar{P}$$

**Figure 4:**
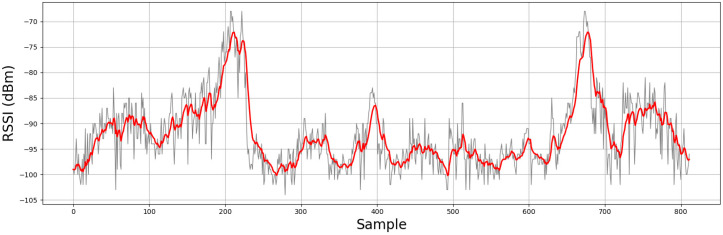
RSSI signal of beacon placed in the door. In gray, the raw RSSI signal. In red, the filtered RSSI signal.

The residual, or innovation (y) equals to the measurement (z) plus the current state ($\bar{x}$). The parameter to be set in this phase is the measurement noise R. This value is the estimated variance of the z measurements. $R={\left(12\;\mathrm{dBm}\right)}^{2}$ was used because it was observed that the RSSI readings fluctuate with a standard deviation of around 12 dBm when the beacon and smartphone are separated at a fixed distance. Figure 4 shows a real example of filtering the received RSSI values for one of the beacons used in the experiment.

### 4.3. Initialization of Beacons’ Positions

In this work, it is only possible to estimate the distance range between the person (smartphone) and the beacon. There is no way to get any orientation information. First, the positions of all beacons are unknown. Assuming that the beacon’s signal propagation pattern is spherical, all points belonging to that sphere with a radius equal to the estimated range are possible points at which the beacon could be located. Consequently, the uncertainty in the position of the beacon, represented in the EKF by the state covariance matrix, tends to be very large, causing non-convergence of the filter. Two measures were taken to address this problem: (1) the establishment of a small distance range to start and update the position of the beacons and (2) the initialization of beacons’ location by using particle filters.

Regarding the first measure, the stability of the RSSI readings is affected by increasing the power of the beacon and also by increasing the distance between the beacon and the receiver. For this reason, the transmission power of the beacons was configured at –20 dBm, which is equivalent to a maximum range of around 3.5 m based on the manufacturer specifications. Additionally, when observing the RSSI readings collected in the dataset, it was found that the RSSI readings are stable up to an approximate range of 2.5 m, equivalent to an RSSI value of −88 dBm. Therefore, if the received RSSI value is less than −88 dBm, that value is not taken into account either for initialization or for updating the beacon location.

#### 4.3.1. Location Initialization for Stationary and Active (Non-Mobile) Beacons

Upon receiving the first RSSI reading higher than −88 dBm, the distance between the beacon and the smartphone (z = radius) is obtained through Equation (5). The measurement noise (R) of z is assumed to be 10% of z. For example, if it is estimated by Equation (5) that the beacon is in a radius of 2 m, the beacon may be on a disk between 1.8 m and 2.2 m. The next step is to create n=2000 uniformly distributed particles inside the disk. For the maximum range allowed, that is, with  z=2.5 m, there is $\;2.54\frac{\mathrm{particles}}{{\mathrm{cm}}^{2}}$. Each particle represents a possible beacon location. The particle distribution was performed as described in Cook [24] using the following equations:

(9) $$x=\sqrt{r}\cos\theta$$

(10) $$y=\sqrt{r}\sin\theta$$

With r∈[radius−(radius×0.1), radius+(radius×0.1)] and  θ∈[0, 2π]. Each particle has an associate weight  (w), which is proportional to the probability of being the particle representing the true beacons’ position. When the first z is estimated, the weights of all the particles are initialized with a value $\;w=\frac{1}{n}$.

##### Prediction

With each new stride detected, the particles must move the amount traveled by the person plus an amount given by the process noise (Q), which was established at$\;0.01\;m^{2}$. The new position of each particle is given by:

(11) $$pos_{x}=pos_{x}+\left(stride_{length_{x}}+rand\times Q\right)\;$$

(12) $$pos_{y}=pos_{y}+\left(stride_{length_{y}}+rand\times Q\right)$$

where randn is a random decimal number and  randn∈[0,1].

##### Update

Starting with the second estimated z, the particle weights are updated using the following equation:

(13) $$w_{t}=w_{t-1}\times p(z|distance,R)$$

With p(z|distance,R) being the probability density function of the measurement z given the distance between the particle and the person and the measurement noise R.

##### Resampling

This phase replaces the particles that have a very low weight, with copies of particles that have a high weight. After having updated the weights of all the particles, the number of effective particles is calculated using:

(14) $$\;{\hat{N}}_{eff}=\frac{1}{\sum_{i=1}^{N}{\left(w^{i}\right)}^{2}}$$

In case ${\hat{N}}_{eff}$ is less than half of the total particles, the sampling process is activated. There are different methods for this purpose, such as multinomial, residual, stratified, and systematic resampling [25]. Stratified and systematic resampling have shown to outperform the other methods in several problems. Systematic resampling ensures a uniformed sampling from all the particle space while ensuring larger weights are proportionality resample more often. Stratified resampling ensures that particles with larger weights being resampled more [26]. Both approaches were tested with the proposed method. As no relevant performance differences were identified, systematic resampling was used for all further experiments.

##### Initial Position Estimate

After the update phase is done, it is possible to estimate the position of the beacon by calculating the sum of the weighted values of the particles:

(15) $$pos_{x}=\frac{1}{N}\;\overset{N}{\underset{i=1}{\sum }}w^{i}x^{i}\;pos_{y}=\frac{1}{N}\;\overset{N}{\underset{i=1}{\sum }}w^{i}y^{i}$$

In the same way, the mean of the variance of all the particles can be estimated. When the value of the variance of both coordinates reaches a value of less than $\;0.01\;m^{2}$, the beacon position initialization phase is finished.

#### 4.3.2. Initialization and Re-Initialization of Mobile Beacons

When a person uses the broom, the pitcher, or the hairbrush, that element changes its position with respect to the x-y coordinates. Also, it likely changes its position with respect to the z coordinate. Furthermore, these items may not be returned to the same point from which they were taken. Consequently, its position has to be reset every time its use is detected. A particle filter was also used for this purpose. The difference with the method described for locating the other beacons is that instead of creating a disk and generating 2000 particles within it, in this case, the upper half of a sphere is built, and 10,000 particles are distributed uniformly into it based on Cook [24]. The measurement noise (R) was set to 10% of z. The external and internal radius of the sphere is given by  r∈[z−R, z+R] and:

(16) azimuth=2π×u

(17) $$polar=\sin^{-1}\left(1-v\right)$$

Variables u and v are random decimal numbers such that u, v∈[0,1]. The polar coordinates of each particle generated with the previous equations were transformed into Cartesian coordinates. The minimum density of particles is reached with the maximum z allowed (2.5 m). For that case, the density of particles is$\;2.53\frac{\mathrm{particles}}{{\mathrm{cm}}^{3}}$. The prediction, update, and sampling phases are the same as those described above. The estimate of the moving beacon’s final position is given when it is detected that the beacon stopped moving. The estimates of the x-y coordinates of that instant are established as the new position of the beacon.

#### 4.3.3. Estimation of the Person and Beacons Position

The estimation of the person’s location is carried out utilizing a particle filter. At the beginning of each session, 600 particles are created. The current state $s^{t}$ of each particle represents a possible x-y position, so the history of the particle’s states makes up a probable trajectory followed by the person$\;S^{t}=\left\{s_{1},\;s_{2},\ldots ,\;s_{t},\;\right\}$. With N = 600, there are 600 possible paths at each time step: $S_{n}^{t}=\left\{s_{1}^{n},s_{2}^{n},\ldots ,s_{t}^{n},\right\}$ such that n∈[1600].

On the other hand, the estimation of the position of the beacons must be done separately for each particle, since the estimation of the position of the beacons depends on the position of the person (Equation (4)). To achieve this, each beacon’s position was estimated with an independent extended Kalman filter (EKF), as proposed by Montemerlo et al. [20].

#### Prediction

When a new stride is detected, the information of its length and direction is sent to the prediction phase, where the new state of each particle is estimated. The equations for this are identical to Equations (11) and (12). The process error Q is assumed to be a fixed value equals to $\;0.01\;m^{2}$; that is, the standard deviation of the stride length is 0.1 m.

#### Update

Once the first estimate of the location of a beacon is obtained through the initialization module, the estimate and its covariance matrix are used as the initial state of the respective EKF. Beacons do not provide orientation information; in consequence, the used EKF implements an update phase that relies only on range information. It was adapted from the range-only SLAM method proposed by Menegatti et al. [27]. The state and covariance matrix of its EKF must be updated when a new distance measurement is received from a beacon. For this, the transition function is given by the distance between the current estimate of the person’s position and the current estimate of the beacon’s position:

(18) $$h_{n}\left(x_{b}^{n},y_{b}^{n}\right)=\sqrt{{\left(x_{s_{t}}^{n}-x_{b}^{n}\right)}^{2}+{\left(y_{s_{t}}^{n}-y_{b}^{n}\right)}^{2}\;}$$

The next step is to calculate the residual, which represents the error between the received distance measurement to the beacon  z, and the estimate made with the transition function:

(19) $$y=z-h\left(\bar{x}\right)$$

Since $h\left(\bar{x}\right)$ is not a linear function, it must be linearized by calculating its Jacobian. The SciPy Python library was used for this purpose. The result is:

(20) $$H=\left[\frac{x_{s_{t}}^{n}-x_{b}^{n}}{h_{n}},\;\frac{y_{s_{t}}^{n}-y_{b}^{n}}{h_{n}}\right]$$

With the calculated residual and Jacobian, the state and its respective covariance matrix can finally be updated using the Kalman filter equations:

(21) $$K=\bar{P}H^{T}{\left(H\bar{P}H^{T}+R\right)}^{-1}$$

(22) $$x=\bar{x}+Ky$$

(23) $$P=\left(I-KH\right)\bar{P}$$

Finally, the weight of each particle is updated:

(24) $$w_{t}^{n}=w_{t-1}^{n}\times p(z|h_{n},R)$$

#### Resampling

**Figure 5:**
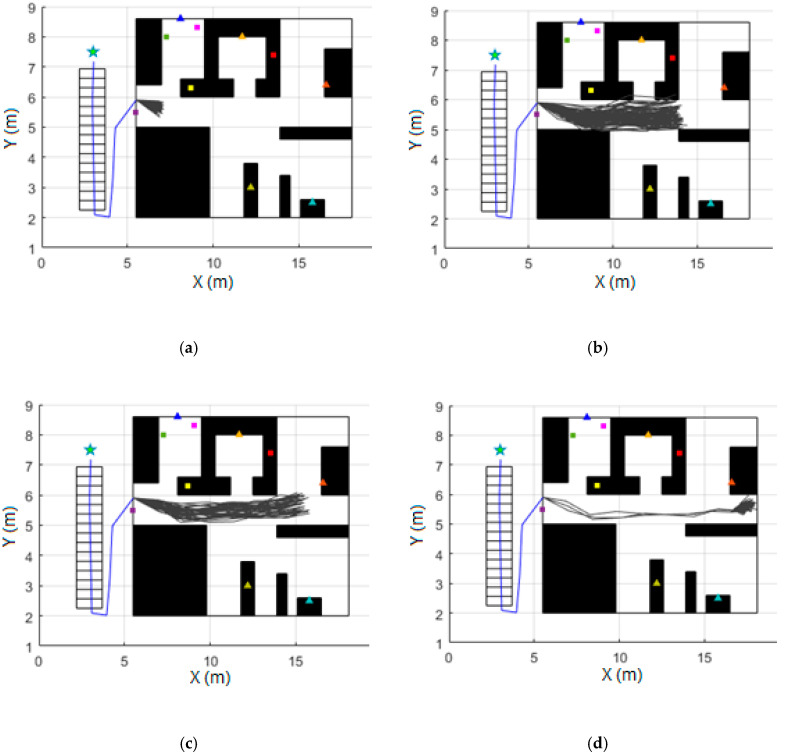
Example of prediction, update, and resampling phases: (**a**) the particle filter is initialized once the movement of the door is detected. 600 new different trajectories (gray lines) are created, and the prediction phase is executed when one stride is detected; (**b**) Possible trajectories just before receiving the first RSSI measurement from the beacon in the room; (**c**) Update and resampling phases are run for the first time when the RSSI measurement is processed; (**d**) Update and resampling phases are run when with the second RSSI measurement received.

Since each of the 600 particles generated has an associated weight representing the probability of tracking the actual person’s trajectory, it is desired that the particles with a very low weight be replaced by others that have a high weight for the filter to converge. After the weight of all the particles has been updated, the number of effective particles ${\hat{N}}_{eff}$ is calculated as described in Section 4.3.1. In case this is less than half of the total particles used (200 for this case), sampling is carried out using the systematic resampling method [25]. An example of the prediction, updated, and resampling phases is shown in Figure 5.

### 4.4. Evaluation

The evaluation process consisted of the trajectory reconstruction (indoor localization) and beacons localization (mapping) for each of the eight participants using the dataset described in Section 3. The software implementation of the proposed method was performed using a combination of Python and Matlab. Its source code, the dataset collected for its evaluation, the code of the mobile application developed to collect the data from the beacons, and the scripts used for the preparation and synchronization of the data are available via the web (see web addresses in the Supplementary Materials).

#### 4.4.1. Evaluation of Beacons Localization—House Mapping

At the beginning of the first activity’s execution, which is to enter the simulated apartment, the position of the beacons is unknown. After the participant enters the radius of coverage of a beacon (around 3.5 m), the initialization process for the localization of the respective beacon begins. Once the beacon position is initialized, subsequent beacon’s RSSI measurements are used to refine the participant’s position using the particle filter. Simultaneously, the beacon’s position is refined with its respective EKF utilizing the participant’s position. The beacon’s localization error is equal to the Euclidean distance (in two dimensions) between the beacon’s ground-truth position and its estimated position.

#### 4.4.2. Evaluation of Pedestrian Indoor Localization

After the position of an active beacon is initialized, it is used to reset the participant’s position when the participant interacts with that beacon. The interaction is inferred to happen when the movement of the beacon is detected. At that moment, the loop is closed, which means that the participant’s location is reset, and the localization error is evaluated. The localization error is calculated as the Euclidean distance (in two dimensions) between the ground truth (real) beacon’s position and the participant’s estimated position.

The total trajectories in all the test have the same starting and ending point. Thus, the localization error was also calculated at the end of each test, using as ground-truth the real coordinates of the starting/ending point.

The prediction phase in the particle filter involves the addition of a random error (Equations (15) and (16)) to produce different filter state options that possibly capture the actual state of the system. Due to the random nature of these errors, the evaluation of the location of the beacons and the participants varies in each execution for the same participant. As a strategy for evaluation, it was decided to execute the process 10 times for each participant and get the average of the 10 runs.

## 5. Results

### 5.1. Beacon Localization—House Mapping

The average localization error of the ten beacons was 0.82 ± 0.24 m. The average localization error of the stationary beacons was 0.96 ± 0.65 m (Table 3) and one of the active beacons was 0.67 ± 0.29 m (Table 4). For the mobile beacons, the actual position was considered as the position before the beacon started moving and the estimated position as the position after the beacon was moving.

**Figure 6:**
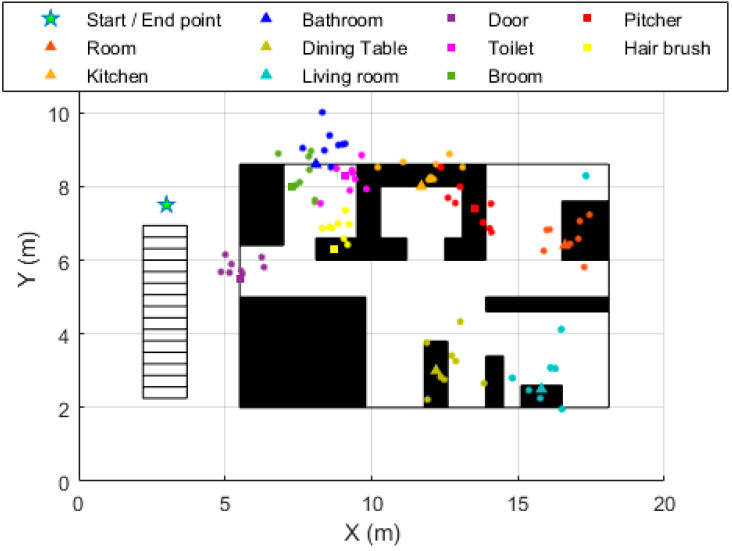
Graphic results of the beacons localization. Points are the beacons’ locations estimates. Each point corresponds to the average of the 10 location estimates of a beacon for each participant. The ground-truth location and the location estimates for each beacon share the same color.

The beacons’ localization results are also presented graphically (Figure 6). The ground-truth and estimated locations of each beacon were drawn with the same color to visualize how much the estimates varied from the actual beacons’ position.

### 5.2. Pedestrian Indoor Localization

The mean of the localization error for all the participants was 1.05 m ± 0.44 m (Table 5). The person’s trajectory is made up of the sum of the estimated locations of each of their strides.

**Figure 7:**
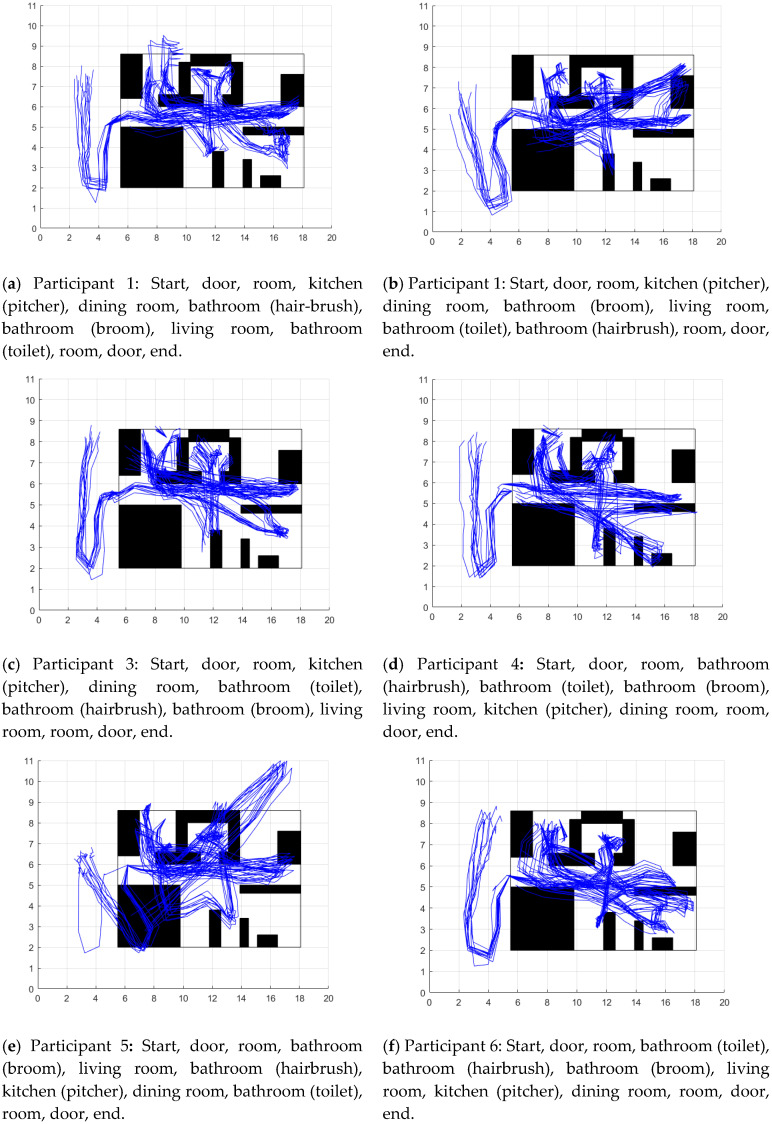

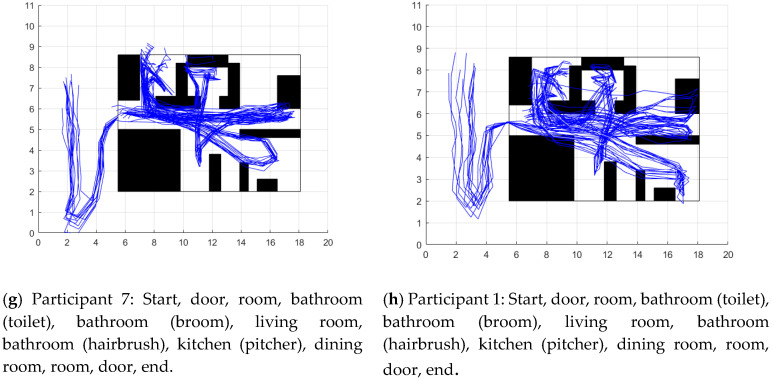
Results of participant’s localization. The blue lines are the 10 trajectories reconstructed for each participant. The description of each image has the sequence followed by the participant.

Figure 7 contains the 10 reconstructed trajectories for each participant. The trajectories were aligned with the map only at the beginning of the first activity taking as a reference to the first participant’s strides while descending the stairs.

## 6. Discussion

The results obtained demonstrate that the proposed method could point to a promising roadmap towards the development of simultaneous localization and home mapping system based only on one IMU and some Bluetooth Low Energy Beacons. To the best of our knowledge, no work has taken advantage of the additional benefits of beacons, including movement detection to improve indoor localization and house mapping using SLAM.

The method involves the use of one IMU at the user’s foot. This enables PDR implementation with Zero-Velocity-Updates. This strategy is recognized for providing higher precision in the calculation of stride size and, therefore, in location tracking compared to others that use the IMU on other parts of the body [11]. Furthermore, the use of the IMU at the foot could be useful for Ambient Assisted Living scenarios, where obtaining gait parameters are useful for the early detection and monitoring of diseases like Parkinson’s disease [28].

The complexity and robustness of an IPS based on the proposed method were fundamental characteristics taken into account in its design. Robustness is an intrinsic characteristic of an IPS based on the SLAM recursive algorithm framework since the location of the person, and the location of the landmarks are continually updated. Therefore, if there is any kind of change in the position of furniture or obstacles, the IPS can adapt to them. Regarding the complexity of the IPS, the proposed method would allow the user to place the beacons at home in the same way that they were used in this work. Without prior knowledge of the environment, the method can obtain a map of the environment, which is described through the location of the beacons. The latter is true, having in mind that the proposed method is aimed at environments such as houses or apartments of a similar size to that used in the evaluation in this work.

### 6.1. Pedestrian Indoor Localization

The average localization error for the eight participants was 1.05 ± 0.44 m. This result is better than those obtained in the related literature [14,15,16,17,18]. However, a direct comparison between the studies is not possible because all the related works were evaluated in different scenarios of different shapes and sizes and using different transmitting/receiving devices. Therefore, we cannot conclude that our proposed method offers better precision. Moreover, the localization error in most of the related works was measured only at one reference point, which is usually the same point at which the person begins to walk. However, in our method, the localization error was measured every time the movement of an active beacon was detected. At the same time, the person’s position was reset to the last established position of that beacon. The shorter the distance traveled by the person, the less the drift suffered due to the estimation of the position with PDR [7], which may be the reason we managed to reduce the error compared to related studies.

It is important to discuss the number of beacons used. The key to our approach is that the person’s position is corrected based on closeness to the beacons. That is why in this work, it was chosen to put a ‘stationary’ beacon in each room of the apartment and active or mobile beacons on objects with which a person usually interacts. One way to reduce the average position error obtained is to increase the number of beacons, as verified in Zuo et al. [18], thereby increasing the number of beacons from 24 to 48, they managed to reduce the position error from 4.69 m to 3.25 m. However, the more beacons used, the more expensive the IPS. The trade-off between cost and precision must be found.

It is also important to highlight that our method’s evaluation protocol included activities in which the person had to remain standing or sitting. These activities make location tracking even more difficult as they lead to stride detection errors and possibly a loss of orientation due to unusual foot movements. The key to handling the possible errors produced by such movements is the fusion of the PDR method’s data with the range of distance to the beacons.

Understanding the person’s trajectory as the person’s location history, the fusion of inertial data with distance from beacons generally allowed to construct the map and to reconstruct the trajectory of the participants (Figure 7). However, the reconstructed trajectory for participant 2 and 5 was not good. An orientation error seriously affected the trajectory. That caused the location of the beacons to be erroneously initialized or updated. Since the mapping and trajectory are continuously updated, this would lead to an exhaustive analysis of how often the person should visit the different places in their home in such a way that the SLAM-based method can correct a large orientation error, such as the one that occurred in the mentioned cases. Instead of deepening on that analysis, there are two possible and more direct solutions: the first is to include in the data fusion the orientation data given by a magnetometer, a sensor with which it is possible to obtain the orientation concerning the magnetic poles of the earth. The second possible solution is to increase the transmission power of the stationary beacons to have more range measurements to correct the error.

The particle filter used to estimate the person’s location used 600 particles, which correspond to 600 possible locations that are evaluated every time a distance measurement to a beacon is available. The greater the number of particles to be updated, the higher the processing capacity necessary to execute the proposed method. In Hardegger et al. [29], an exhaustive analysis of the relationship between the number of particles and the processing time required for updating and resampling was carried out. They obtained that 1000 particles were sufficient to achieve adequate precision in such a way that it was possible to run the set of their SLAM algorithms on a smartphone. In our case, an analysis of the precision of the location concerning the number of particles was performed using 10 quantities of particles starting at 100 and ending at 1000. The result indicates that with 600 particles, the precision of the location reaches a maximum value. In this way, it could be said that it is very likely that the proposed method can be run on a smartphone.

### 6.2. Beacons Localization—House Mapping

The map of the house or apartment is represented by the deployed beacons. Therefore, the beacons localization process is essential, not only to obtain a graphic description of the environment but also to complement the information of the PDR method and correct possible errors caused by the IMU.

A total of ten beacons were used, five of them were categorized as stationary. Its purpose was to indicate proximity to distinct parts of a home, such as a kitchen, bathroom, living room, dining room, and bedroom. The localization error of the stationary beacons was 0.96 ± 0.65 m. Bearing in mind that a maximum radius of distance allowed between the beacon and the participant was established as 2.5 m, the average error obtained is equivalent to 38.4% of the aforementioned maximum range. This result demonstrates the difficulty in locating a Bluetooth beacon even using low transmission power and filtering the signal with a Kalman filter. The effects of the multipath phenomenon on Bluetooth signals are the cause of the difficulty of estimating the distance between the beacon and the receiving device using the Log-distance path model. Some authors have suggested that the use of RSSI is not suitable for applications in a real environment [30]. However, we believe that the results obtained were sufficient for the proposed method to describe the environment map (Figure 7) and simultaneously locate the person within the map.

The average localization error of the active beacons was less than that of the stationary beacons (0.67 ± 0.29 m). One possible justification for this result is that more RSSI values were received from active beacons than from stationary beacons. This makes sense because the participants interacted with the active beacons and for that reason, they were closer and longer compared to the stationary beacons. That allows the particle filter to converge quickly by processing more reliable range measurements.

The process for locating the active beacons attached to the door and the bathroom was the same as for the stationary beacons, that is, 2000 particles were uniformly distributed in a disk with an upper radius equals to z+(z×0.1) and a lower radius equal to  z−(z×0.1), where z is the estimated distance between the beacon and the person calculated using the path loss model equation (Equation (1)). The remaining three beacons were considered mobile beacons. For them, 10,000 particles were uniformly distributed in the upper half of a sphere with an upper and lower radius calculated in the same way as for the disk. Although the number of particles per unit area was similar when the disk or sphere was used, the error of the mobile beacons’ localization was greater in all cases compared to the active non-mobile beacons (Table 4). Increasing the number of particles for the mobile beacons’ localization could reduce the error obtained, but this would depend on the maximum threshold of the processing capacity of the device in which the proposed method would be executed.

A previous study deployed 48 beacons uniformly in an indoor environment [18]. They demonstrated that the greater the number of beacons deployed in the environment, the smaller the error of their location. When using 48 beacons, the error was 1.27 m, and using 24; the error increased to 2.26 m. Although the average localization error of the 10 beacons used in our work was 0.82 ± 0.24 m, the area of the evaluation environment in Zuo et al. [18] was 24 times greater than the area of the environment used in our work. For that reason, although the average error obtained by our proposed method is less, it cannot be established that it improves the result obtained in Zuo et al. [18].

Based on the previous analysis, it is suggested to carry out a simple training phase to obtain a more precise location of the beacons. That phase would consist of the person addressing each beacon deployed in the environment and staying as close as possible to each beacon for at least ten seconds so that the particle filter converges by processing enough measurements taken at a short distance and, therefore, more reliable one.

## 7. Conclusions

In this study, we have proposed and evaluated a novel method for Simultaneous Indoor Localization and house mapping, reaching a localization error of 1.05 ± 0.44 m, and a mapping error of 0.82 ± 0.24 m. The pedestrian movement was captured from a foot-mounted IMU. The position estimates were obtained by a PDR algorithm that used the pedestrians’ movement data. The position estimates were improved by fusing them with proximity and activity-related data from BLE beacons deployed in the indoor environment.

The data fusion was performed using the fast-SLAM algorithm, which was originally designed to track the robot’s position through a particle filter. At the same time, the algorithm simultaneously estimates the position of various landmarks using EKFs. In our study, the BLE beacons were used as landmarks that provided activity-related events and estimates of the distance to the pedestrian. Since, in the beginning, the location of the beacons is not known, our method implements one step to initialize those positions.

To the best of our knowledge, this is the first method that includes the beacons’ data movement as activity-related events in a method for pedestrian Simultaneous Localization and Mapping (SLAM). The results are promising, and further evaluations in different scenarios should be carried out to ensure the proposed method can map them and get the pedestrian localization. Enabling the reception of the Bluetooth signal from beacons in the IMU would be an important step towards deploying an IPS method in a real environment. In this way, the use of the smartphone for solely receiving Bluetooth signals from the beacons would be avoided. If the beacon signal is received by the IMU placed on the foot, relocating the stationary beacons at ground level should be considered to get a line of sight and simplify the distance estimation.

The final objective of our work is to take advantage of the relationship between human activity and the indoor location where it occurs, that is, that the human activity recognition is based on location information and that the indoor localization is supported by the human activity recognition. In this article, we have focused on indoor localization. Therefore, our next step will be directed towards the human activity recognition using IMU data together with the data related to activities collected from the beacons. In this way, the location errors obtained might be reduced. We will use the proposed method in ambient assisted living scenarios with the aim of tracking both activities and the location of elderly people. Therefore, in a future evaluation, data will be collected from older adults, and the use of different apartments or houses will be included. The number of beacons concerning the size of the place will also be considered.

## Figures and Tables

**Table 1:** Related works.

Reference	SLAM Landmarks	Testbed Size (Approx.)	Number of Beacons	Precision
[14]	Detection of corners	20 m × 30 $m^{2}$	NA	1.34 m
		70 m × 70 $m^{2}$	NA	0.8 m
[15]	Detection of activities	10 m × 10 $m^{2}$	NA	1.16 ± 0.07 m
[16]	RFID tags	30 × 40 $m^{2}$	NA	2 m
[17]	WIFI routers	60 m × 10 $m^{2}$	NA	2.23 ± 1.25 m
[18]	Bluetooth beacons	90 m × 37 $m^{2}$	48	3.25
			24	4.69

Approx: Approximately; NA: Not Applicable.

**Table 2:** Activities performed by the participants.

Main Activities	Sub-Activities
Enter the apartment	Go down the stairs, go to the door and open it.
Take off jacket	Go into the room and leave the jacket there.
Serve something to eat and go to the dining room to eat.	Go to the kitchen and serve some drinks (using the pitcher) and take something to eat. Go to the table and eat what was served.
Sweep	Go to the bathroom, take the broom, go to the living room, and sweep it. Return the broom to the place where it was taken.
Combing	Go to the bathroom, take the comb, and comb your hair.
Use the toilet	Go to the bathroom, lift the seat cover that simulates the toiler lid, and sit in it.
Leave the apartment	Go for the jacket left in the room. Put on the jacket and leave the house. Finish the activity at the same starting point.

**Table 3:** Localization error of stationary beacons.

Participant	Bathroom	Kitchen	Room	Living Room	Dining Room
**1**	1.42	0.30	0.18	0.65	0.72
**2**	0.52	0.40	0.83	1.75	0.67
**3**	1.14	0.78	0.49	0.73	0.23
**4**	0.62	1.57	0.73	0.88	0.83
**5**	0.92	0.90	0.74	5.98	1.67
**6**	0.47	0.46	0.88	0.25	0.36
**7**	0.91	1.30	0.67	1.03	0.82
**8**	1.06	1.50	1.18	0.43	1.56
**Average error (m)**	0.88 ± 0.30	0.9 ± 0.47	0.71 ± 0.27	1.46 ± 1.75	0.86 ± 0.48

Each value is the average localization error of the 10 runs.

**Table 4:** Localization error of active beacons.

Participant	Door	Hair Brush	Broom	Toilet	Pitcher
**1**	0.48	0.57	0.11	0.78	0.49
**2**	0.15	0.70	0.27	0.81	0.59
**3**	0.81	0.49	1.01	0.26	0.75
**4**	0.21	0.45	0.73	0.44	1.59
**5**	0.94	0.86	1.14	0.35	0.76
**6**	0.66	1.12	0.85	0.25	0.87
**7**	0.88	0.67	0.98	1.13	0.65
**8**	0.38	0.61	0.86	0.34	0.93
**Average error (m)**	0.56 ± 0.28	0.68 ± 0.20	0.74 ± 0.34	0.55 ± 0.30	0.83 ± 0.31

Each value is the average localization error of the 10 runs.

**Table 5:** Average localization error for each participant (ten runs).

Participant	Error (m)
1	0.79 ± 0.28
2	1.24 ± 0.56
3	1.16 ± 0.49
4	1.34 ± 0.52
5	1.38 ± 0.85
6	0.95 ± 0.34
7	0.71 ± 0.26
8	0.77 ± 0.26
Average	1.05 ± 0.44

## Supplementary Materials

Python project for data preprocessing including the raw and synchronized dataset available at: https://github.com/jesusceron/DataPreProcess; Android application for beacons data collection available at: https://github.com/jesusceron/BeaconsDataReceiver; Matlab-Python source code for pedestrian SLAM available at: https://github.com/jesusceron/pedestrian_SLAM and https://github.com/jesusceron/IL_HAR_app_Matlab.

## Appendix A

### SLAM Background

In the context of human indoor localization, the objective of SLAM is to simultaneously obtain a map of the indoor environment and the location of the person on that map. The person’s position at a time t ($s_{t})$ depends on the previous position $s_{t-1}$ and the movement measurement $u^{t}$ that describe a change of position. For example, a measurement of a movement sensor indicating that the person has moved a certain distance. Therefore, $s_{t}$ is described as a probability density function known as the motion model:

(A1) $$p(s_{t}\;|\;u_{t},\;s_{t-1}),$$

The map of the indoor environment is represented by landmarks. A landmark can be observed explicitly, such as the radio frequency signal coming from a device, or implicitly by detecting elements of the environment such as corridors, stairs or corners. In general, each landmark is associated with a specific point in the indoor environment. In probabilistic terms, the probability density function with which the position of the landmarks is estimated is known as the measurement model:

(A2) $$p(z_{t}\;|\;s_{t},\;\Theta ,n_{t}),$$

with $z_{t}$ being the distance between the person and the landmarks detected at time t. $\Theta \;=\left\{{\Theta \;}_{1},\;{\Theta \;}_{2},\;\ldots ,\;{\Theta \;}_{t}\;\right\}$ is the set of all the landmarks and $n_{t}\;$ is an array with the IDs of the detected landmarks. The vector $n_{t}$ is known as the correspondence since it relates each landmark with a specific identifier. Sometimes it is not possible to uniquely identify each landmark initially, for example, if the system receives the signals from different sources without any type of identification. In that case, the identification of each landmark must be made as an extra step before or during the execution of SLAM.

As a result, the SLAM problem applied in the context of human indoor localization can be posed as the problem of simultaneously determining the position of the person and of all landmarks established in the indoor environment by using the measurements $u_{t}$ and $z_{t}$. Assuming that the correspondence vector is known, the probability density function that described this is:

(A3) $$p(s^{t},{\Theta \;}^{t}\;|\;z^{t},\;u^{t},n^{t}),$$

where $s^{t}=\left(s_{1,}s_{2},\;\ldots ,s_{t}\right)$ is the set of person’s positions, $z^{t}=\left(z_{1,}z_{2},\;\ldots ,z_{t}\right)$ y $u^{t}=\left(u_{1,}u_{2},\;\ldots ,u_{t}\right)$ are the set of measurements and $n^{t}=\left(n_{1,}n_{2},\;\ldots ,n_{t}\right)$ the set of landmarks detected in each time step.

The location estimation of each landmark n ϵ NL=1,2,…,n is an independent localization problem. Therefore, Equation (A3) can be factorized as follows:

(A4) $$p(s^{t},\;\Theta \;|\;z^{t},\;u^{t},n^{t})=p(s^{t}|\;z^{t},\;u^{t},n^{t}){\prod^{\;}}_{n=1}^{NL}\;p{(\Theta \;}_{n}\;|\;s^{t},\;z^{t},u^{t},n^{t}),$$

Equation (A4) shows how the problem of locating the person and the landmarks at the same time can be addressed by NL+1 estimators: one to estimate the person’s trajectory and *NL* to estimate the location of the set of landmarks.
